# The Congress Report

**Published:** 1893-10

**Authors:** 


### THE CONGRESS REPORT.

We present our readers in this number with papers selected from the large number read at the Columbian Dental Congress. Arrangements were made prior to this meeting to give a continued report of the proceedings, but finding this would result in very little profit to our readers, the present course was adopted as being the most satisfactory. All of the papers selected for this and the succeeding number will bear careful reading, and several of them are of rare scientific value.

